# Preventing sexually transmitted and blood borne infections (STBBIs) among sex workers: a critical review of the evidence on determinants and interventions in high-income countries

**DOI:** 10.1186/s12879-019-3694-z

**Published:** 2019-03-05

**Authors:** Elena Argento, Shira Goldenberg, Kate Shannon

**Affiliations:** 1 Department of Medicine, University of British Columbia, Centre for Gender & Sexual Health Equity, 1190 Hornby Street, Vancouver, BC V6Z 2K5 Canada; 2 Interdisciplinary Studies Graduate Program, University of British Columbia, 2357 Main Mall, Vancouver, BC V6T 1Z4 Canada; 3 Faculty of Health Sciences, Simon Fraser University, 8888 University Drive, Burnaby, BC V5A 1S6 Canada; 4 School of Population and Public Health, Faculty of Medicine, University of British Columbia, 2206 East Mall, Vancouver, BC V6T 1Z9 Canada

**Keywords:** Sex workers, HIV prevention, STBBI, Risk environment, High-income countries, Structural interventions

## Abstract

**Background:**

Across diverse regions globally, sex workers continue to face a disproportionate burden of HIV and other sexually transmitted and blood borne infections (STBBIs). Evidence suggests that behavioural and biomedical interventions are only moderately successful in reducing STBBIs at the population level, leading to calls for increased structural and community-led interventions. Given that structural approaches to mitigating STBBI risk beyond HIV among sex workers in high-income settings remain poorly understood, this critical review aimed to provide a comprehensive synthesis of the global research and literature on determinants of HIV and other STBBIs and promising intervention practices for sex workers of all genders in high-income countries.

**Methods:**

We searched for publications over the last decade (January 2005–March 2016) among sex workers (cis women, cis men, and trans individuals). Data obtained from quantitative peer-reviewed studies were triangulated with publicly available reports and qualitative/ethnographic research where quantitative evidence was limited.

**Results:**

Research demonstrates consistent evidence of the direct and indirect impacts of structural factors (e.g., violence, stigma, criminalization, poor working conditions) on increasing risk for STBBIs among sex workers, further compounded by individual and interpersonal factors (e.g., mental health, substance use, unprotected sex). Sub-optimal access to health and STBBI prevention services remains concerning. Full decriminalization of sex work has been shown to have the largest potential to avert new infections in sex work, through reducing workplace violence and increasing access to safer workspaces. Promising practices and strategies that should be scaled-up and evaluated to prevent STBBIs are highlighted.

**Conclusions:**

The high burden of STBBIs among sex workers across high-income settings is of major concern. This review uniquely contributes to our understanding of multilevel factors that potentiate and mitigate STBBI risk for sex workers of all genders. Research suggests that multipronged structural and community-led approaches are paramount to addressing STBBI burden, and are necessary to realizing health and human rights for sex workers. Given the heterogeneity of sex worker populations, and distinct vulnerabilities faced by cis men and trans sex workers, further research utilizing mixed-methods should be implemented to delineate the intersections of risk and ameliorate critical health inequalities.

## Background

In many high-income countries and regions, such as Canada, the United States, Australia, and New Zealand, sex workers are amongst key populations most heavily affected by the HIV epidemic and continue to face a disproportionate burden and elevated risk for HIV and other sexually transmitted and blood borne infections (STBBIs) [1, 2]. Some examples of other STBBIs include chlamydia, gonorrhea, syphilis, and hepatitis C (HCV). Global estimates of STBBI burden among sex workers remain challenged by poor surveillance, research methods, and limited data; however, the overall prevalence of HIV among female sex workers has been estimated to be 11.8% in lower and middle-income countries (LMICs) [3] and 1.8% in high-income countries [1, 4]. The overall HIV prevalence rate among the general population in high-income countries has been estimated to be 233 per 100,000 population [5]. Structural and community-level factors continue to both increase STBBI vulnerabilities (e.g., violence, stigma) and mitigate acquisition (e.g., safer working conditions, sex worker-led programming) among sex workers [1, 2]. Such factors account for the significant heterogeneity in burden of STBBIs among sex workers within and across settings.

Sex workers represent a diverse population including cis women, cis men, and trans individuals. The organization of sex worker populations varies considerably by work setting [2, 6], with sex workers operating out of more formal in-call establishments (e.g., massage parlours, beauty salons, brothels), out-call or other informal indoor venues (e.g., bars, hotels, lodges, saunas), and outdoors (e.g., streets, parks, public spaces). Sex workers solicit clients in equally diverse settings: independent self-advertising (e.g., online, newspapers, or by phone/text), through escort agencies or in-call venues (e.g., massage parlours), or through a third party (e.g., manager; book keeper) [2, 6]. The majority of sex workers are cis female with cis male clients; however, cis male and trans sex worker populations exist in many settings, including with cis female patrons [7, 8]. Cis or cisgender refers to a gender identity that aligns with the physical sex assigned at birth, while trans is an umbrella term that represents transgender and transsexual, but may also include gender diverse and “two spirit” individuals. The term “two spirit” is often used among Indigenous people to refer to someone with both feminine and masculine spirits, and has a fluid, non-binary meaning [9, 10]. Of note, data are especially sparse among trans men; therefore the available data among trans sex workers is limited to trans women [8].

In recent years, research and evidence demonstrate that behavioural and biomedical interventions alone are only moderately successful in reducing STBBIs and increasing condom use, emphasizing the urgent need to scale-up structural interventions and community-led programs [1, 2, 11], which focus on reducing social and economic inequalities by addressing larger social, legal, and policy barriers. Structural community-led interventions are committed to ensuring health and human rights of sex workers and are driven by the needs and priorities of sex workers themselves; for example, community-led programs have successfully implemented workshops with establishment owners/managers to facilitate commitment to HIV prevention across sex work venues and drop-in centres for sex workers and their intimate partners, including educational and holistic sex health services [12, 13]. In the context of criminalization of sex work, policing and stigma/discrimination (e.g., from police, healthcare providers) continue to dissuade and prevent sex workers from carrying condoms and hinder any efforts made to increase access to health and support services, including provision of antiretroviral therapy (ART) to sex workers. In recent years, multipronged structural approaches to STBBI prevention largely based in LMICs are being considered globally as models of best practice, acknowledging the significance of structural determinants of risk for sex workers, including interpersonal relationships with clients and intimate/non-paying partners and the importance of understanding the larger legal and policy environments alongside biology and individual behaviours [1, 2, 14].

Numerous research and medical experts, international bodies, and sex work communities around the globe have formally endorsed decriminalization of sex work, given the well-established evidence that criminalized and enforcement-based approaches to sex work have harmful impacts. The first ever WHO/UNAIDS international guidelines on HIV/STI prevention, treatment and care among sex workers (published in 2012 with the Global Network of Sex Work Projects [NSWP]), prioritizes removal of all criminal laws targeting sex work as a necessary approach to ensuring the health, safety, and human rights of sex workers [11]. International bodies, including Global Commission on HIV and the Law, United Nations Development Program (UNDP), United Nations Population Fund (UNFPA), and Amnesty International have all strongly endorsed the evidence-based need for decriminalization of sex work [11, 15, 16].

Despite efforts to consider structural determinants in STBBI prevention interventions, such as community empowerment approaches in many settings in the global north, and Canada in particular [17], stigma, criminalization, and a lack of funding continue to impede large-scale implementation. In 2014, the leading medical journal, the *Lancet,* published a special issue on “HIV and Sex Work,” launched as a major session at the World AIDS conference [1, 2, 7, 8]. A series of papers in the Lancet systematically reviewed the evidence and determinants of vulnerability and interventions to prevent HIV among sex workers and released an urgent call to action to address the disparities and drivers of high burden and suboptimal HIV prevention, treatment, and care among sex workers globally [2]. Determinants and structural approaches to mitigating STBBI risk beyond HIV among sex workers in high-income countries remain poorly understood. Drawing on a structural determinants framework – one that aims to elucidate the role of intersecting social, structural, and environmental factors in shaping individual-level health outcomes [2, 18, 19] – this aim of this critical review is to provide a comprehensive synthesis of the global literature and evidence on HIV and other STBBI vulnerability and promising prevention practices for sex workers of all genders in high-income settings.

## Methods

A literature search was conducted of publications over the last decade (January 2005 – March 2016) on STBBIs among sex workers (cis women, cis men, and trans individuals) in the 27 high-income countries classified by the World Bank (OECD members) in 2016 [20]. Publications were assessed for the extent to which they reported on STBBIs and condom use outcomes, as well as structural, individual, and biomedical determinants and interventions in the mitigation or potentiation of STBBI acquisition and transmission risk.

### Search strategy

This review triangulates information and data obtained from searching peer-reviewed reports published in English in key databases: PubMed (MEDLINE), Social Sciences Citation Index, Science Citation Index Expanded, Arts & Humanities Citation Index, and Emerging Sources Citation Index (via Web of Science). The following search terms were used in combination and connected with “and”: **sex work terms** (“sex work*” OR “sex worker” OR “prostitut*” OR “prostitution” OR “commercial sex worker*” OR “transsexual” OR “cross dresser” OR “transvestite” OR “escort” OR “gay for pay”); **HIV/STBBI-related terms** (“HIV” OR “human immunodeficiency virus” OR “HIV infections” OR “AIDS” OR “acquired immunodeficiency syndrome” OR “acquired immune deficiency syndrome” OR “condom use” OR “non-condom use” OR “condom non-use” OR “unprotected sex” OR “condom refusal” OR “condom negotiation”, “condom utilization”, “sexually transmitted infection*”, “STI”, “blood borne infection*” OR “STBBI” OR “STD” OR “sexually transmitted disease*” OR “hepatitis C” OR “HCV” OR “chlamydia” OR “gonorrhea” OR “syphilis”); **risk and intervention-related terms** (“risk factor*” OR “correlate” OR “determinant” OR “predictor” OR “risk” OR “HIV risk” OR “risk behavior*” OR “risk behaviour*” OR “intervention*” OR “empowerment” OR “community-based” OR “treatment” OR “prevention” OR “strateg*” OR “structural approach*”); **terms for high-income countries** (“Canada” OR “United States” OR “USA” OR “US” OR “North America” OR “England” OR “United Kingdom” OR “UK” OR “Australia” OR “New Zealand” OR “Europe” OR “Spain” OR “Italy” OR “Germany” OR “France” OR “Finland” OR “Denmark” OR “Norway” OR “Sweden” OR “Austria” OR “Switzerland” OR “Belgium” OR “Portugal” OR “Netherlands” OR “Holland” OR “Japan” OR “Czech Republic” “Greece” OR “Hungary” OR “Iceland” OR “Ireland” OR “Korea” OR “Luxembourg” OR “Slovak Republic”). EA and SG did initial screening and EA extracted relevant data and information from each study (i.e., country, study design, population, STBBI and condom use outcomes, individual/interpersonal and structural determinants and interventions) and relevant reports.

The search was supplemented with additional sources, including publicly available reports (e.g., Open Society Foundation, NSWP, WHO and UN reports), and qualitative/ethnographic peer-reviewed research where quantitative evidence was limited.

### Inclusion and exclusion criteria

We included quantitative studies that examined risk factors for STBBIs (e.g., HIV, HCV, chlamydia, gonorrhea or syphilis) or condom use (including condom refusal and unprotected sex) among cis female, cis male, and trans sex workers in high-income countries. Non-primary research (e.g., commentaries), studies without full-text sources available (e.g., abstracts), studies in which STBBIs or condom use were not analyzed as outcomes, studies that did not report multivariable analyses, and those that did not stratify results by sex workers were excluded. Qualitative studies and publicly available reports were included to provide further context around quantitative work. We excluded studies that focused solely on adolescents (< 18 years), studies from LMICs, and non-English publications.

## Results

### Individual and interpersonal determinants

To date, most research on sex work and STBBIs has focused on the experiences of female sex workers. Despite reports on the high prevalence of STBBIs among both cis men and trans sex workers [21–24], little data exist on the determinants of STBBIs unique to these populations. Current evidence suggests the determinants of STBBIs for sex workers of any gender are highly multifaceted. Individual and interpersonal determinants (including substance use and sexual risk practices, mental health, and partner-level variables, among others) were examined in 12 studies focusing on female sex workers (Table 1), 3 studies focusing on cis male sex workers (Table 2), and 3 studies focusing on trans sex workers (Table 3).

**Table 1:** Studies describing determinants associated with STBBI-related outcomes among cisgender female sex workers in high-income countries

Study Details				Outcome Examined		Multivariable Associations Reported	
Author/Year	Country	Study Design/Dates	Population	STBBI Outcome	Condom Use Outcome	Individual & Interpersonal Determinants	Structural Determinants
Argento et al., 2014 [29]	Canada (Vancouver)	Cross-sectional 2010–2013	369 female sex workers (trans inclusive)		Inconsistent condom use with intimate partners	Inconsistent condom use was positively associated with having a cohabiting (aOR 5.43, 95%CI 2.53–11.66) or non-cohabiting intimate partner (aOR 2.15, 95%CI 1.11–4.19) (versus casual partner), providing drugs (aOR 3.04, 95%CI 1.47–6.30) or financial support to an intimate partner (aOR 2.46, 95%CI 1.05–5.74), physical intimate partner violence (aOR 2.20, 95%CI 1.17–4.12), and an intimate partner providing physical safety (aOR 2.08, 95%CI 1.11–3.91); non-injection drug use was inversely associated (aOR 0.32, 95%CI 0.17–0.60)	
Argento et al., 2015 [61]	Canada (Vancouver)	Cross-sectional 2010–2013	654 female sex workers (trans inclusive)		Client condom refusal		Social cohesion had an independent protective effect on client condom refusal (aOR 0.97, 95 %CI 0.95–0.99)
Cohan et al., 2006 [96]	USA (San Francisco)	Cross-sectional 1999–2004	783 sex workers (419 female, 187 male, 126 trans)	Prevalence of STI: gonorrhoea (12.4%), chlamydia (6.8%), syphilis (1.8%), or herpes simplex virus 2 (34.3%)		STIs were positively associated with African American ethnicity (aOR 3.3, 95%CI 1.3–8.3), male gender (aOR 1.9, 95%CI 1.0–3.6), and work-related violence (aOR 1.9, 95%CI 1.1–3.3)	STIs were inversely associated with working collectively (aOR 0.4, 95%CI 0.1–0.9)
Deering et al., 2013 [77]	Canada (Vancouver)	Cross-sectional 2010–2011	490 female sex workers (trans inclusive)		Being offered or accepting more money for sex without a condom	Offered and accepting more money for sex without a condom was positively associated with being a sexual minority (aOR 2.72, 95%CI 1.35–5.46), less than daily crystal meth (aOR 2.95, 95%CI 1.27–6.87), speedball injection (aOR 6.93, 95%CI 1.60–29.94), having more clients per week (1.03, 95%CI 1.01–1.06), clients have other sex worker partners (1.83, 95%CI 1.19–2.84), and client violence (aOR 2.18, 95%CI 1.10–4.34)	Offered and accepting more money for sex without a condom was inversely associated with soliciting for clients in indoor settings (aOR 0.15, 95%CI 0.04–0.54)
Goldenberg et al., 2014 [31]	Canada (Vancouver)	Cross-sectional 2010–2011	508 female sex workers (trans inclusive)	Prevalence of HIV (11.2%), combined STI/HIV (20.9%)		HIV infection was positively associated with early sex work initiation: < 18 years vs. 18+ years (aOR 2.49, 95%CI 1.35–4.64), < 16 years vs. 16+ years (aOR 1.88, 1.03–3.42)	
Goldenberg et al., 2015 [38]	Canada (Vancouver)	Prospective cohort 2010–2013	715 female sex workers (trans inclusive)	HCV prevalence (43.6%); HCV incidence (4.28 events per 100 person-years)		HCV incidence was inversely associated with age (aHR 0.91, p = 0.04), and positively associated with STI co-infection (aHR 3.45, *p* = 0.04), and crack use (aHR 4.24, *p* = 0.05); HIV co-infection was also positively associated with incidence of HCV in a separate model.	
Kweon et al., 2006 [39]	Korea	Cross sectional Jan-July 2004	1527 female sex workers (HIV negative, non-IDU)	HCV prevalence (1.4%)		HCV was positively associated with history of acupuncture (aOR 3.3, 95%CI 1.16–9.34) and diabetes (aOR 11.2, 95%CI 2.63–47.8)	
Lee et al., 2010 [36]	Korea	Cross-sectional June-Nov 2008	999 female sex workers	Prevalence of chlamydia (12.8%)	Condom use last night; last month	Higher prevalence of chlamydia was positively associated with younger age and higher inconsistency of condom use.	
Mc Grath-Lone et al., 2014 [83]	England	Cross-sectional Jan-Dec 2011	2704 female sex workers	Prevalence of HIV (0.2%), syphilis (0.1%), chlamydia (10.1%), gonorrhoea (2.7%), HCV (0.2%)			Being a migrant sex worker vs. UK-born was inversely associated with prevalence of chlamydia (aOR 0.59, 95%CI 0.46–0.79)
Platt et al., 2011 [30]	England (London)	Cross-sectional 2008–2009	268 female sex workers (indoor-working)	Prevalence of HIV (1.1%), syphilis (2.2%), chlamydia or gonorrhoea (6.4%)		STBBI prevalence was positively associated with age 23–36 years vs. 17–22 years (aOR 12.3, 95%CI 1.44–105.1) and having an intimate partner (aOR 3.0, 95%CI 1.03–8.73)	STBBI prevalence was positively associated with having no contact with outreach services (aOR 3.6, 95%CI 1.14–10.5)
Shannon et al., 2007 [25]	Canada (Vancouver)	Cross-sectional 2004	198 female sex workers (trans inclusive)	HIV prevalence (26%)		HIV infection was positively associated with early (< 18 years of age) sex work initiation (aOR 1.8, 95%CI 1.3–2.2), Aboriginal ethnicity (aOR 2.1, 95%CI 1.4–3.8), daily cocaine injection (aOR 2.2, 95%CI 1.3–3.5), daily crack smoking (aOR 2.7, 95%CI 2.1–3.9), and unprotected sex with intimate partner (aOR 2.8, 95%CI 1.9–3.6).	
Shannon et al., 2009 [60]	Canada (Vancouver)	Cross-sectional Apr-Sept 2006	205 female sex workers (trans inclusive)		Pressured into unprotected sex by client	Client condom refusal was positively associated with sharing a crack pipe with client (aOR 2.5, 95%CI 1.06–2.49) and client violence (aOR 2.08, 95%CI 1.06–4.49)	Client condom refusal was positively associated with working in outdoor/public spaces (aOR 2.00, 95%CI 1.65–5.73), having a workplace zoning restriction from prior charges (aOR 3.39, 95%CI 1.00–9.36), and policing-related displacement (aOR 3.01, 95%CI 1.39–7.44)
Sou et al., 2015 [85]	Canada (Vancouver)	Cross-sectional 2010–2013	182 migrant female sex workers (trans inclusive)		Inconsistent condom use by client		Inconsistent condom use by clients was positively associated with difficulty accessing condoms (aOR 3.76; 95%CI 1.13–12.47); and inversely associated with servicing clients indoors (aOR 0.34, 95%CI 0.15–0.77) and education (aOR 0.22, 95%CI 0.09–0.50)
Surratt et al., 2012 [49]	USA (Miami, Florida)	Cross-sectional 2006–2010	562 female sex workers (drug users)		Unprotected vaginal sex	Unprotected sex was positively associated with age (aOR 1.03, 95%CI 1.01–1.05) and client violence (aOR 1.82, 95%CI 1.22–2.72)	
Surratt et al., 2012 [35]	USA (Miami, Florida)	Cross-sectional 2007–2010	562 female sex workers (drug users)	HIV prevalence		HIV prevalence was positively associated with early initiation into sex work before age 18 (aOR 2.10, 95%CI 1.25–3.54)	HIV prevalence was positively associated with history of foster care (aOR 3.68, 95%CI 1.62–8.35)

**Table 2:** Studies describing determinants associated with STBBI-related outcomes among cisgender male sex workers in high-income countries

Study Details				Outcome Examined		Multivariable Associations Reported	
Author/Year	Country	Study Design/Dates	Population	STBBI Outcome	Condom Use Outcome	Individual & Interpersonal Determinants	Structural Determinants
Fournet et al., 2016 [22]	Netherlands	Cross-sectional 2006–2012	3053 male sex workers	Prevalence of HIV (2.5%), STI (18.1%; syphilis, chlamydia, gonorrhoea)		HIV+ status was positively associated with younger age (aRR 2.74, 95%CI 1.15–6.50), and sexual minority (aRR 24.41, 95%CI 3.37–176.88); Bacterial STIs were positively associated with younger age (aRR 2.30, 95%CI 1.83–2.88), sexual minority (aRR 1.62, 95%CI 1.27–2.06), previous STI in past 2 years (aRR 1.39, 95%CI 1.15–1.68), and HIV+ status (aRR 2.71, 95%CI 1.68–2.64)	HIV+ status was positively associated with not having a previous HIV test (aRR 2.59, 95%CI 1.56–4.29)
Grov et al., 2015 [52]	USA	Cross-sectional	387 male sex workers (internet-based escorts)		Unprotected anal sex with last client and last non-client	Condomless anal sex with last client was positively associated with depression (aOR 1.13, 95%CI 1.02–1.25); Condomless anal sex with last non-client was positively associated with HIV+ status (aOR 12.29, 95%CI 1.56–96.92)	
Mc Grath-Lone et al., 2014 [88]	England	Cross-sectional Jan-Dec 2011	488 male sex workers	Prevalence of HIV (3.7%), syphilis (2.6%), chlamydia (24.7%), gonorrhoea (17.4%)			Being a migrant male sex worker vs. UK-born was positively associated with prevalence of chlamydia (aOR 2.20, 95%CI 1.08–4.49)
Sethi et al., 2006 [21]	England (London)	Cross-sectional 1994–2003	823 male sex workers	HIV prevalence (9.3%); HIV incidence (49 cases)		HIV infection was positively associated with injection drug use and unprotected anal sex with casual partner	HIV incidence was positively associated with first attending the clinic earlier, in 1994–1996 vs. 1997–1999 (*p* = 0.007) or vs. 2000–2003 (*p* = 0.02)

**Table 3:** Studies describing determinants associated with STBBI-related outcomes among trans sex workers in high-income countries

Study Details				Outcome Examined		Multivariable Associations Reported	
Author/Year	Country	Study Design/Dates	Population	STBBI Outcome	Condom Use Outcome	Individual & Interpersonal Determinants	Structural Determinants
Clements-Nolle et al., 2008 [23]	USA (San Francisco)	Cross-sectional	190 trans women sex workers		Inconsistent condom use with clients (receptive anal sex)	Inconsistent condom use with clients was positively associated with low self-esteem (aOR 3.09, 95%CI 1.28–7.47), a history of forced sex/rape (aOR 2.91, 95% CI 1.06–8.01), and crack-cocaine use (aOR 2.59, 95%CI 1.09–6.13)	
Dias et al., 2015 [40]	Portugal	Cross-sectional Jan-Sept 2011	1040 sex workers (81 trans, 106 male, 853 female)	HIV prevalence (17.6% among trans; 7.4% among female; 5% among male)		HIV infection was positively associated with older age (over 25 compared to 18–25), trans identity (aOR 6.35, 95%CI 1.66–24.26), and ever use of psychoactive drugs (aOR 4.06, 95%CI 2.16–7.67)	HIV infection was positively associated with lower (<€1000) monthly income (aOR 2.62, 95%CI 1.16–5.91) and working outdoors (aOR 5.43, 95%CI 1.90–15.56)
Nemoto et al., 2014 [24]	USA (San Francisco and Oakland)	Cross-sectional 2000–2001 & 2004–2006	573 trans women (53% with history of sex work)		Unprotected receptive anal sex with clients and non-paying partners	Unprotected anal sex with commercial partner was inversely associated with norms towards healthy behaviors (aOR 0.39, 95%CI 0.15–0.98), and self-efficacy toward safe sex (aOR 0.27, 95%CI 0.10–0.76); Unprotected anal sex with primary partner was inversely associated with Latina ethnicity (aOR 028, 95%CI 0.09–0.88) norms towards healthy behaviors (aOR 0.51, 95%CI 0.31–0.85), and self-efficacy toward safe sex (aOR 0.48, 95%CI 0.25–0.91); Unprotected anal sex with primary partner was positively associated with depression (aOR 2.58, 95%CI 1.24–5.38)	Unprotected anal sex with commercial partner was positively associated with exposure to transphobia (aOR 2.56, 95%CI 1.12–5.87)

While the HIV epidemic among sex workers is predominantly driven by sexual transmission [2, 7, 25], sex workers who inject drugs are at elevated risk for HIV/STBBIs through dual drug and sexual risk pathways. Among female sex workers in Europe, injecting drugs is the primary individual-level determinant of HIV [26]. A recent systematic review estimates 35–65% of female sex workers in the USA use injection drugs, and reported rates of crack use were as high as 75% [27]. In a recent study of 1647 people who inject drugs in Vancouver, sex workers who injected drugs had a significantly higher cumulative incidence of HIV than other injection drug users (12% vs. 7%); multivariable analyses suggest that HIV risk for sex workers who inject drugs appears to be modified by contextual factors and dual sexual and risks linked to daily cocaine injection [28].

Dual drug and sexual risk behaviours significantly enhance risks for STBBI transmission among sex workers and their partners. Studies of interpersonal determinants of STBBIs have begun to identify how types of partners (e.g., regular or one-time clients and intimate/non-paying partners) shape sexual risks and STBBI transmission dynamics. For example, condoms are less likely to be used in transactions with regular clients as compared to one-time clients, and similar to estimates in the general Canadian population, nearly three-quarters of female (trans inclusive) sex workers in Canada report recent inconsistent condom use with intimate partners [29]. Among indoor-working cis female sex workers in the UK, having an intimate partner was significantly associated with prevalence of HIV and other STBBIs [30].

In Canada, Indigenous sex workers (i.e., First Nations, Metis, Inuit ancestry) are more than twice as likely to be living with HIV than their non-Indigenous counterparts, and being younger, substance using, and reporting unprotected sex were all associated with increased risk of STBBIs [25, 31–33]. A Los Angeles based study among cis female and trans women sex workers identified African/American ethnicity, higher income, cohabitation, and not seeking recent health care as predictors of lower HIV prevention utilization [34]. While the vast majority of sex workers initiate sex work in adulthood, duration of time in sex work has been shown to shape risk pathways for STBBIs, including increased risk for HIV infection among women sex workers in Vancouver and Miami [31, 35]. Among cis female sex workers in Korea, younger age and higher inconsistency of condom use were associated with chlamydia infection [36]. Male sex workers (MSWs) in Europe report injecting drugs significantly longer than other men who inject drugs, with a higher proportion sharing needles (23% vs. 14%), and MSWs reported incarceration at a significantly younger age than their non-sex worker counterparts [37]. In the Netherlands, younger men were more than twice as likely to test positive for STBBIs, and identifying as gay or bisexual significantly increased risk [22].

Despite dual drug and sexual risks, little is known about the burden of HCV among sex workers. One study found elevated risk for HIV-HCV co-infection among sex workers in Canada: close to half (44%) of sex workers in the study had HCV, with higher odds among women who reported having a recent STI, being HIV positive, Indigenous ancestry, engaging in sex work longer, older age, and working outdoors [38]. The HCV incidence density was 4.28 events/100 person-years, with highest rates among sex workers who inject drugs, and multivariable analysis demonstrated both STI and non-injection stimulant crack use to be major pathways to HCV infections [38]. HCV prevalence among cis female sex workers who do not inject drugs in Korea was positively associated with history of acupuncture and diabetes [39].

Globally, trans women sex workers can have up to six times increased odds of HIV infection than cis male or cis female sex workers, with injection drug use being highly prevalent among those living with HIV [40, 41]. Limited Canadian research highlights the wide range of sexual risks and heterogeneity of trans individuals; in Ontario, only half of trans women (of which 15% had a history of sex work) were ever tested for HIV [42].

Among MSWs, risks for STBBIs are driven by a range of unique biological and structural factors and this population has seen a sustained and possibly increasing burden of HIV/STBBI globally [1, 43]. The primary risks for STBBIs identified among MSWs include unprotected anal intercourse, having a high number of sexual partners and large sexual networks, and stigma [7, 44–47]. Some evidence suggests that a higher proportion of MSWs report condom use than gay men or other men who have sex with men (MSM) who do not exchange sex: 51% vs. 30%, respectively [44]. In Ontario, MSWs were more likely to test positive for HIV and HCV-HIV co-infection than MSM not involved in the sex industry [48]. Among the limited available data among MSWs, research from the UK found that rates of both unprotected sex and gonorrhoea both increased steadily over the study period (1994–2003) [21]. Among MSWs in the Netherlands, the rate of STIs (syphilis, chlamydia, gonorrhoea) increased from 15.2 to 21.1% between 2006 and 2010 and then dropped to 18.3% in 2012 [22].

Sex workers in Canada and the USA report high levels of childhood trauma and violence, and among those with histories of violence and abuse, mental illness may be mediating the association between trauma and unprotected sex [29, 49]. Estimates of mental health issues among female sex workers vary significantly across aspects of the industry and settings, ranging from low levels to as high as 74% reporting severe depression, anxiety or post-traumatic stress [49]. Evidence suggests historical experiences of violence and indirect violence (i.e., witnessing violence) can contribute to STBBI risk by shaping the propensity to use drugs for self-treatment of emotional trauma [50].

Comorbid substance use and mental health problems among sex workers may elevate risk for violence and risk of STBBIs from partners to sex workers [45, 46, 51]. A recent study identified depression as significant predictor of unprotected anal sex with the most recent male client among MSWs who work online [52]. The majority (91%) of North American MSWs reported having sex while drunk; 32% had a history of depression; 41% had a history of childhood sexual abuse; and 79% of those who work on the street had been incarcerated [45]. One-third reported being HIV positive and one-quarter had never been tested for STIs [45]. In the UK, one-fifth of MSWs surveyed reported a history of mental illness and 21% reported a history of sexual abuse, with multivariable analyses demonstrating significant associations between injection drug use and HIV [21].

A USA-based systematic review found a high prevalence of sex work involvement among trans women, with an estimated 24–75% of trans women reported to have participated in sex work in their lifetime [53]. Among trans sex workers, low self-esteem and a history of rape was independently associated with inconsistent condom use [54], and the majority of trans women (of whom 53% reported sex work in the last 6 months) surveyed in the USA reported suicidal ideation or attempts and depression [55].

### Structural determinants

#### Violence against sex workers

Epidemic rates of physical, sexual, and verbal violence against sex workers continue to be reported globally and have among the strongest links to elevated STBBI burden among sex workers [56]. Violence – whether by clients, individuals posing as clients, police officers, strangers, or exploitative managers or pimps – reduces or eliminates sex workers’ ability to control their working conditions and safely negotiate terms of transactions (e.g., types of sex acts and whether condoms are used) placing sex workers at elevated risks for STBBI acquisition [57]. In environments where sex work is criminalized, physical and sexual violence is the most pervasive and influential determinant of HIV and other STBBI risk among sex workers; the evidence is clear that physical/sexual violence is associated with inconsistent condom use, client condom refusal, and STBBI risk [58–61].

Cities across Canada are sadly home to epidemics of violence against sex workers, with Indigenous and street-involved sex workers facing the highest burden, and yet there continues to be a lack of coordinated response. A systematic failure to protect women sex workers from violence over decades has led to deeply-rooted mistrust of health and service providers [62, 63]. Research has shown that avoidance of health services in Vancouver, Canada due to violence, fear of violence, and negative interactions with police displaces marginalized sex workers to more isolated spaces – particularly youth and Indigenous women [64, 65].

#### Criminalization and enforcement-based approaches

In high-income settings, criminalization, incarceration, and legal restrictions have consistently been directly linked to elevated risks for HIV/STBBI acquisition through increased risk of violence and abuse [60, 66, 67]. The evidence is unequivocal that in criminalized settings, sex workers are forced into adversarial relationships with police officers and are unable to access essential social, health, and legal protections [2, 68]. Police have used possession of condoms as evidence of sex work to justify arrest, which creates a substantial disincentive among sex workers to use protection with clients [57, 66, 69], and is a gross violation of human rights. Condoms have also been used as evidence to target third parties and sex work businesses, which directly effects access to condoms.

For sex workers in Sweden, structural stigma and the law have led to increased violence and social exclusion, including housing instability [69]. Police surveillance and harassment (e.g., enforced displacement to isolated areas, detainment without arrest, threated or enacted violence or coercion) in criminalized settings directly influences the ability to negotiate condom use and types of sexual practices with clients by forcing sex workers to rush transactions, forge screening prospective clients, and displacing workers to more isolated/hidden venues where the risk of violence from clients is greatly elevated [6, 70, 71]. The 2013 landmark *Bedford* ruling in Canada that ultimately struck down Canada’s criminalized sex work laws in the unanimous decision by the Supreme Court [72], was based heavily on robust evidence and science demonstrating direct and indirect harms of criminalization and policing on sex workers health, safety, and human rights.

#### Stigma and discrimination

Stigma and discrimination continue to increase STBBI vulnerability for sex workers. Fear of disclosure of sex work status or drug use to family, friends, and service providers has been both quantitatively and qualitatively linked to increased barriers to health care for sex workers of all genders [73, 74] and increased risk of HIV and HCV [75]. Punitive policies that perpetuate stigma and discrimination against sex workers have been associated with an increase in economic and social insecurity (e.g., homelessness, social isolation), as well as inconsistent condom use [2, 61, 76]. Stigma and discrimination are major barriers to reporting violence to authorities, and result in increased violence and victimization for sex workers [68, 77, 78].

Social stigma, homophobia and transphobia create environments that are especially hostile for trans sex workers and greatly undermine health and safety. Stigma is associated with poverty, refugee or migrant status, ethnicity, substance use, and healthcare avoidance among trans individuals globally [8, 79]. Legal restrictions and confusion of gender-appropriate identification create further barriers to accessing social and health care services in some settings, thus potentiating the economic reliance on sex work and risks for STBBIs. In California, unprotected anal sex with clients and intimate partners was significantly associated with transphobia, economic pressure, HIV/STI co-infection, and identifying as homosexual [24]. Qualitative work from Vancouver and San Francisco shed light on the ways in which different trajectories of risk and violence are shaped by socio-structural factors, such as transphobia and criminalization, with trans sex workers experiencing complex and multilayered vulnerability to STBBIs based on their identity, ethnicity, class, and appearance [78, 80]. Narratives of men and trans sex workers reveal highly diverse gender and sexual identities, underscoring the need to address homophobia/transphobia and reduce stigma and violence for these populations [81]. Qualitative work with MSWs in New York exemplifies how experiencing discrimination and medical distrust can impede access to biomedical HIV prevention strategies such as PrEP (pre-exposure prophylaxis) [74].

#### Migration and mobility

While overall burden of STBBI among international im/migrant sex workers from non-endemic settings has been shown to be lower than locally-born sex workers in high-income settings (e.g., Canada, UK) [82, 83], substantial gaps remain in accessing safe, non-judgemental health care that can impede and potentiate risk for STBBIs [59]. Evidence suggests that im/migrant sex workers face persistent and unique challenges to sexual health and safety, including cultural and language barriers, elevated human rights violations, and fear and distrust of immigration, police, and health service providers [84–86]. In high-income settings, short-term internal mobility or migration (e.g., movement within regions and countries) has been linked to higher burden of STBBI through disrupted social networks and supports, reduced control over working conditions, and elevated risks for violence. Internal mobility and migration for sex work in Canada has been linked to gaps in health services, including disruptions in ART [87]. Among MSWs in England, being a migrant worker vs. UK-born was positively associated with a two-fold increased odds of chlamydia [88]. Mobile/migrant women sex workers in Canada were more likely to be younger, work in indoor in-call establishments, and earn higher incomes, suggesting that short-term mobility for sex work and migration increase social and economic opportunities [89]. However, mobility and migration were also linked to partner condom refusal and reduced health care access, and mobility was associated with enhanced workplace sexual/physical violence, suggesting that mobility/migration may confer HIV and other STBBI risks through less control over work environments and isolation from health and support services.

#### Suboptimal access to STBBI testing and care

Sex workers continue to experience suboptimal access and barriers to STBBI testing and care, and consistently experience structural barriers to safe, non-judgemental health care. Among marginalized sub-populations of im/migrant and Indigenous sex workers, access remains even worse [2, 87], with heightened stigma and discrimination, fear of violence, and language and cultural barriers. There are extremely limited data on ART use and care experiences among sex workers living with HIV in high-income settings, despite being a key population affected by the epidemic [90]. Structural barriers to scaling up and retention in ART remain a major challenge among sex workers globally [1, 11]. Recent data from Canada suggest that incarceration and mobility/migration are major barriers to access and retention of ARTs among sex workers living with HIV [87]. While there is limited research on access to HCV care among sex workers, a recent study from Vancouver estimates nearly 50% of sex workers have not accessed testing for HCV in the past year, and recent immigrants to Canada were less likely to have accessed testing compared to Canadian-born sex workers [91].

### Interventions and promising practices to prevent STBBIs

Studies describing context of promising structural-level intervention and prevention practices to reduce STBBIs among sex workers in high-income countries and policy implications are outlined in Table 4.

**Table 4:** Studies describing promising structural-level intervention and prevention practices among sex workers in high-income countries

Reference	Country	Study Design / Methods	Population	Context of Promising Structural-Level Intervention and Prevention Practices	Policy Implications
Abel et al., 2012 [101]	New Zealand	Survey and qualitative interviews	58 sex workers (all genders)	Decriminalization & Safer Work Environments. In context of decriminalization of sex work, risk perception influenced workers’ decisions to operate in street-based, managed or private sectors of the sex industry.	Alongside decriminalization, social and economic policies are required to address risk and develop enabling environments across sex work sectors of sex work industry.
Anderson et al., 2015 [86]	Canada	46 qualitative interviews	Migrant/immigrant women (trans inclusive) sex workers and managers/owners of indoor establishments	Decriminalization & Safer Work Environments. Women described how policing practices and licensing requirements for indoor sex work establishments shape violence and conflict with clients.	Removing prohibitive municipal licensing and legislation reform is needed to improve safety of sex work environments.
Argento et al., 2016 [106]	Canada	61 qualitative interviews	Cis and trans men who buy and/or sell sex	Community Empowerment & Safer Work Environments. Community-based project; narratives describe how gentrification and online sex work shape social networks, safety, and control.	Critical need to include voices of men and trans sex workers in policy discussions. Supports decriminalization of sex work.
Cohan et al., 2006 [96]	USA	Cross-sectional	783 sex workers (all genders) accessing care at peer-based clinic (St. James Infirmary)	Community-led Programming & Integrative Care. Sex worker-led, free medical clinic provides substantial care to sex workers of all genders.	Sex worker-led and integrative, non-judgmental health and support services are key to reducing STBBIs.
Kim et al., 2015 [98]	Canada	Cross-sectional 2010–2013	547 street-involved women (trans inclusive) sex workers accessing women-only drop-in service	Community-led Programming & Integrative Care. Sex worker-specific drop-in service had high uptake (60% used services in last 3 years), associated with increased access to sexual and reproductive health services.	Low-threshold and sex work-specific models for sexual health should be scaled-up.
Krusi et al., 2012 [76]	Canada	39 qualitative interviews & 6 focus groups	Marginalized women (trans inclusive) sex workers living/working in low-barrier, supportive housing for women	Safer Work Environments. Unsanctioned indoor sex work environments in the context of supportive housing programs increased sex workers’ control over negotiating transactions and condom use with clients.	Removing social and legal barriers to women-only supportive housing models are critical to facilitate safer indoor sex work environments.
Krusi et al., 2014 [71]	Canada	31 qualitative interviews and ethnographic observation	Street-involved women sex worker (trans inclusive)	Decriminalization. Criminalization of sex work and policing practices targeting clients increase risk of HIV/STBBIs.	Decriminalization of sex work is needed to ensure health and human rights for sex workers.
Lyons et al., 2015 [78]	Canada	Qualitative interviews	33 trans women sex workers	Decriminalization & Safer Work Environments. Transphobia and criminalized approaches to sex work shape violence and safety with clients and police.	Need for legal reform of sex work laws and culturally competent anti-stigma programs/policies to reduce transphobia.
Matthen et al., 2016 [81]	Canada	Qualitative interviews	45 men and trans sex workers and clients	Community-led Research. Narratives revealed highly diverse gender and sexual identities, underscoring importance of giving voice to gender and sexual minority sex workers through community-based research.	Policies and services must reflect diversity and needs of sex workers. Critical need to address homophobia/transphobia and reduce stigma.
Mimiaga et al., 2008 [45]	USA	Survey and qualitative interviews	31 MSM sex workers (19 street-involved and 13 internet-based escorts)	Safer Work Environments. Narratives highlight contextual differences in sexual risk-taking among street vs. internet-based workers. 69% reported unprotected serodiscordant sex.	Need for tailored interventions that acknowledge heterogeneity of sex workers and contextual and psychosocial factors influencing workplace safety.
Parsons et al., 2007 [109]	USA	Qualitative interviews	46 male sex workers (internet escorts)	Community Empowerment. Highlights the individual and community needs of male escorts.	Importance of addressing community-identified needs beyond safer sex, such as support with business and legal advice.
Reisner et al., 2008 [51]	USA	Brief survey and qualitative interviews	32 male sex workers	Integrative Care. Findings highlight valuable intervention components: trauma-informed mental health and substance abuse treatment, access to HIV/STI testing and treatment services, support groups to address isolation/loneliness, skill-building for risk reduction with partners, and paid incentives as add-ons to behaviour change interventions.	Multipronged interventions to reduce sexual risk-taking are needed for male sex workers, including addressing unique socioeconomic and legal needs.
Sausa et al., 2007 [80]	USA	Focus groups	48 trans women (85% had ever engaged in sex work); ethnic minorities	Community Empowerment. Participation in sex work and risks were influenced by social networks, cultural norms, immigration, racism, and transphobia	Highlights unique needs of trans sex workers who are ethnic minorities. Further research and polices must be tailored to this key subpopulation.
Shannon et al., 2008 [70]	Canada	Participatory-based focus groups	46 marginalized women sex workers (trans inclusive)	Safer Work Environments & Decriminalization. Lack of safe working environment and policing displace sex workers and elevate risk of violence and STBBI. Peer networks improve safe sexual practices with clients.	Socio-structural environment plays key role in shaping drug and sexual risk of HIV. Need for safer work environment supported by legislative reform.
Underhill et al., 2015 [74]	USA	31 qualitative interviews	Male sex workers	Decriminalization. Narratives highlight how experiencing discrimination and medical distrust can impede access to biomedical HIV prevention strategies such as PrEP.	There is a need to address multiple stigmas and discrimination that create barriers to STBBI prevention.
Williams et al., 2006 [108]	USA	Questionnaires to evaluate brief interventions to increase condom use	112 street-based male sex workers	Safer Work Environments & Integrative Care. Two-thirds of men enrolled in a brief risk reduction intervention completed it. Condom use during paid sex increased post-intervention.	Brief interventions tailored to male sex workers to reduce unprotected anal sex with clients are acceptable and efficacious.
Whitaker et al., 2011 [75]	Ireland	Qualitative interviews	31 female and 4 male sex workers (drug users)	Decriminalization & Integrative Care. Sex workers described experiencing stigma and discrimination from healthcare providers, which increased risk of HIV and HCV.	Training for service providers is needed to change language and reduce stigma around sex work.

#### Sex worker-led programming and community empowerment

Community empowerment, a process by which sex workers take collective ownership of programs to achieve the most effective outcomes and address social and structural barriers to health and human rights [13], can be a powerful factor in mitigating STBBI among sex workers. However, despite decades of grassroots organizing among sex workers in Canada, there is an astonishing dearth of data on community empowerment in high-income countries, with available global data largely restricted to low and middle-income settings, namely India and Brazil. Criminalization, stigma and a lack of funding to scale up efforts continue to impede progress in many settings to implement large-scale community empowerment efforts to prevent STBBIs [13]. Sex worker-led and community empowerment-based approaches in LMICs place emphasis on organization at the community level (e.g., sex worker drop-in and health services; sex worker-led outreach; peer support; sex work taskforces) to enable sex workers to participate within social and political spheres and protect their own health at the individual level [92]. The defining features of community empowerment among sex workers are that they are community-led, committed to ensuring health and human rights, recognize sex work as work, and driven by the needs and priorities of sex workers themselves [13].

The sex worker-led Sonagachi project in Kolkata, India [93], is perhaps the most well renowned community-based structural approach to HIV prevention. The Sonagachi led to substantial increases in condom use between sex workers and their clients and significantly decreased STI transmission through community awareness and empowerment. Sonagachi is a model for best practices in various other sex worker communities, namely the Avahan/Ashodaya collective, which has combined sex worker-led outreach, advocacy to police and local government, and enhanced sexual health services tailored to sex workers and their partners [94]. Sex worker engagement with police, public, and other community stakeholders (e.g., managers, healthcare providers, government officials) has the potential to alter the risk environment for sex workers by addressing stigma and violence in the industry.

In San Francisco, the St James Infirmary was established as a peer-based occupational health and safety clinic for sex workers of all genders and operates within a harm reduction framework of sex worker-led programming (one-third to one-half of staff are experiential) [95]. The St James Infirmary provides integrated care to sex workers across sexual and reproductive health, mental and physical health, and includes a number of support services and advocacy efforts. The program is considered a best practice by WHO/UNAIDS, with high update of STBBI testing, treatment and care, as well as linkage to health and support services [96].

In Canada, grassroots sex worker organizing led to one of the largest charter challenge cases at the Supreme Court (*Bedford*) that ultimately struck down criminalized sex work laws [72]. Research has demonstrated that sex worker-led outreach and peer support are critical interventions, increasing access to HIV and other STTBI testing and care. Sex worker mobile and peer outreach services that “meet women where they are at” remain critical low-threshold models to increasing engagement in services for women and promoting connections and referrals to health and support services [97, 98]. Sex worker-led and mobile outreach have been independently linked with increased access to HIV testing and addiction treatment, while sex worker-only drop-in spaces have been linked to greater uptake of sexual and reproductive health care [97, 98]. A pilot intervention of peer-mediated support has also shown increased engagement and retention in care for sex workers living with HIV [99]. Among street and off-street sex workers in Vancouver, higher levels of social cohesion (i.e., mutual support, trust and solidarity) among workers within their work venues or outdoor spaces have been shown to have a direct and independent effect on reducing client condom refusal [61]. Collectively, research highlights the critical need to increase investment and support in community organization and sex work-led programming in the response to prevent STBBIs.

#### Decriminalization

In the *Lancet,* the decriminalization of sex work (i.e., removal of all laws targeting the sex industry including sex workers, clients, and third parties) has now been demonstrated to have the largest potential to reduce HIV infections in sex work, estimated to avert 33–46% of new HIV infections among sex workers and clients in Canada, India and Kenya over the next decade [2]. A number of regions, most notably New Zealand and in some states in Australia, have decriminalized all aspects of sex work, and research by governments and academics have shown increased access to occupational health and safety standards, and better coverage of health services [100, 101], with no evidence of unintended harms. Importantly, WHO/UNAIDS international guidelines, alongside the Global Commission on HIV and the Law and Amnesty International, all call for evidence-based decriminalization of sex work as necessary to prevent and treat HIV. Unfortunately, new legislation in Canada, known as the “Protection of Communities and Exploited Persons Act” (Bill C-36; implemented in December 2014), further criminalizes most aspects of the sex industry, including clients, third parties, self-advertising spaces [102], and evidence suggests this approach perpetuates the same harms through isolating sex workers and reducing ability to control transactions or access to health, social, and legal protections [71].

#### Integrative care tailored to sex workers

Low access to STBBI testing and cervical screening [91, 103] underscores the need for novel structural and sex worker-led approaches to remove barriers to safe, non-judgemental testing and care along the STBBIs. As noted, sex worker-led and mobile outreach have been evidenced to be critical strategies to reaching hidden street and off-street sex workers and building linkages to STBBI prevention and care [84, 97, 103]. Low-threshold sex worker drop-in spaces have been shown to increase access and referral to sexual and reproductive health [98]. The potential to integrate sex work-tailored health care within existing spaces (e.g., drop-in centres, peer support, housing), where sex workers are comfortable and have established connections with community partners, offers a key opportunity for redressing past mistrust and trauma in STBBI health services. Culturally-tailored, language appropriate, and sex worker-led services for mobile and new im/migrant sex workers remain critical to supporting health, safety and access to STTBI prevention and care for this population.

#### Safer work environments

Work environments, as a product and interplay of laws, policies and other structural factors, can both facilitate vulnerabilities to STBBIs or act as critical interventions in supporting sex workers’ health and safety. The work environment refers to physical, social, political and economic features of spaces where sex workers operate. As described previously, exposure to unsafe working conditions, including isolated street and indoor spaces, have been consistently linked to elevated violence, client condom refusal, and other risks for STBBIs [2, 7]. In contrast, access to safer indoor work environments globally have been consistently shown to play a key intervention role in prevention of STBBIs by supporting sex workers’ ability to control transactions, screen prospective clients, and negotiate safer sex transactions and condom use [2]. Longitudinal research in Vancouver, Canada demonstrates that access to safer indoor work spaces (e.g., in-call spaces, massage parlours), with supportive policies and practices (e.g., supportive managerial and venue-based practices) and access to prevention and harm reduction onsite (e.g., bad date report sheets, condoms, lube), increased sex workers’ ability to work together and was linked to reduced risks for violence, non-condom use with clients, and lower STBBI risks [56, 77]. Access to indoor work spaces that promote sex workers’ ability to screen prospective clients, negotiate safety measures, and access health and harm reduction resources remain critical to the health and safety needs of sex workers, including prevention of STBBIs [61, 104].

Evaluation of a novel supportive and women-only housing model for sex workers demonstrates the potential of structural and community interventions to prevent violence and increase safety, including prevention of STBBIs, for the most marginalized sex workers [76, 86, 98]. Qualitative studies and narratives of sex workers demonstrate that access to safer indoor work venues enables sex workers to move away from street-based settings and better control the conditions of work, including connecting with social and legal supports and increasing capacity to refuse unwanted service requests and avoid violent perpetrators [76, 86]. Supportive licensing that allow access to safer indoor work spaces (e.g., locked doors to prevent robberies) and allow managers/third parties to provide resources, hold promise for promoting safer sex work spaces and a number of municipalities in Canada and other settings have taken steps towards more progressive approaches (e.g., City of Vancouver Sex Work Taskforce) [86].

The rise of social media and online platforms has transformed the structure and organization of the sex industry [105]. Recent qualitative research conducted with cis men and trans sex workers and clients in Vancouver highlights how the shift to online solicitation has increased safety and control over the work environment by enhancing screening of prospective clients (e.g., via webcams), increasing sex workers control over transactions and reducing the risk of violence, stigma, and police harassment for both workers and clients [106]. Alongside increased efforts to provide sexual health education and referral and self-testing for some STBBIs across the general population and among gay and other MSM, there represents a critical opportunity for safe, non-judgmental health and support services and peer-led interventions for sex workers through online means. Unfortunately, in the context of new legislation in Canada (PCEPA) that criminalizes buying and advertising of sex, including in online venues and third parties [102], these policies have serious implications for the health and safety of sex workers, many of whom have transitioned to online advertisement and solicitation.

#### Special considerations for Cis men sex workers

Interventions must recognize the heterogeneity of MSWs [7, 45, 107]. Risk reduction is impeded by criminalization of sex work and stigma. While brief risk reduction interventions have been demonstrated to be efficacious in reducing unprotected anal sex with clients among street-based MSWs in the USA, further research is warranted given the heterogeneity of MSW populations [108]. Qualitative research with MSWs has elicited the needs of MSWs who identified important interventions and areas of interest beyond safer sex, such as support with businesses and legal advice [109]. Addressing the specific needs of MSWs requires laws and public health policies that facilitate accessible STBBI prevention and treatment for men, further research to understand context-specific risks, and comprehensive care programs (e.g., willingness/interest to use of PrEP and rectal microbicides). Increasing access to condoms is a necessary but insufficient method on its own [1, 107].

#### Special considerations for trans sex workers

There is a paucity of literature and evidence-based interventions among trans sex worker populations, globally. Behavioural change and biomedical interventions for trans sex workers are promising for preventing HIV in certain settings (e.g., San Francisco), yet ultimately these approaches will not be successful without addressing the upstream drivers of risk [8]. Access to STBBI prevention and other health care services are severely hampered by challenges related to sexual and legal identities, transphobia, and human rights violations. No interventions thus far have ever been developed specifically for trans sex workers.

## Discussion

This critical review of the research over the last ten years demonstrates consistent evidence of the direct and indirect impacts of structural factors (e.g., violence, stigma, criminalization, poor working conditions) on increasing risks for STBBI acquisition among sex workers in high-income countries, building upon evidence from LMICs. Structural factors play a driving role in potentiating and mitigating risk for STBBIs, affecting individual and interpersonal determinants (e.g., mental health, co-morbidities, unprotected sex, substance use) in iterative ways [110]. WHO/UNAIDS international guidelines on HIV/STI prevention, treatment and care for sex workers provide critical recommendations on structural and community-led approaches [11]. In the context of limited understanding of promising practices to mitigate STBBI risk beyond HIV among sex workers in high-income settings, findings from this review highlight shared concerns with evidence from LMICs and the critical need to implement structural and community/sex worker-led strategies globally.

Sub-optimal access to STBBI prevention and care remains detrimental to sex workers across diverse settings worldwide, and evidence suggests this can only be addressed through multipronged, structural and community-led interventions in tandem with biomedical interventions. Of concern, there is limited research documenting sex workers’ experiences of barriers and outcomes to biomedical interventions, particularly ART and PrEP. Access to biomedical interventions (e.g., voluntary testing, ART) alongside community-led approaches has been shown to be instrumental in engaging sex workers in STBBI prevention and care; yet in both North America and Europe STBBI prevention inadequately addresses the psychosocial needs of sex workers and few evidence-based addictions and mental health services are tailored to the needs of sex workers who use drugs [26, 27, 111, 112].

Similar to findings in LMICs, the global evidence among high-income countries suggests that multipronged structural and community-led interventions are urgently needed to increase access to STBBI prevention and care for sex workers [11, 14, 57, 67, 113, 114]. At the macro-level, full decriminalization of sex work now endorsed by WHO, UNAIDS, UNDP, UNFPA, Global Commission on HIV and the Law and Amnesty International, has been shown to have the largest potential to avert HIV infections in sex work, through reducing violence, police harassment, and access to safer work spaces [2]. Meanwhile, evidence indicates that criminalized approaches to sex work reduces access to critical social and health support services, and entrenches individuals in cycles of social exclusion, violence, incarceration, substance use, and poor mental health, infringing upon the human rights of sex workers [16, 68, 69].

At local and regional levels, important evidence has demonstrated the role of safer work environments in reducing risks for STBBIs among sex workers through supportive managerial and venue-based practices, access to harm reduction and prevention resources, and referrals to health and support services [2, 6, 86]. Supportive women-only housing models in Vancouver, Canada have provided a novel intervention approach to ensure the most marginalized sex workers have access to safer indoor work spaces and were linked to increase control over negotiations of sexual risk reduction [76]. As of currently, many of these interventions are small, operate in a legal limbo, and resources to scale-up and further evaluate are urgently needed.

Evidence from both LMICs and high-income settings identified in this review highlight that community and sex worker-led interventions (e.g., peer support, peer and mobile outreach, drop-in spaces) provide a critical window to reaching and providing low-threshold support to sex workers by “meeting people where they are at” and have been linked in the literature to increased uptake of HIV testing, ART, sexual and reproductive health, and addictions treatment, as well as reduced risks for violence [13, 93, 95, 99]. Given these associations, there exists important evidence-based potential to integrate health services within or alongside enhanced drop-in and mobile/peer-led outreach services. Community and sex worker-led strategies that aim to reduce social stigma and health provider discrimination towards sex workers have been shown to have substantial promise elsewhere (e.g., India) and are necessary to ensuring active engagement of the sex work community [2, 13, 93]. While a number of municipalities in Canada and the USA have made some progress through city-wide taskforces towards addressing stigma and violence against sex workers (e.g., licensing reforms to protect sex workers, public education) including police-sex worker dialogues, limited research documents the impacts and socio-legal barriers continue to hamper the ability to fully realize and scale-up potential changes. Sex work-tailored occupational health and safety services with integrated care have shown to be highly effective at engaging sex workers in STBBI prevention and care, and St James Infirmary (USA) offers a promising UN/WHO best practice [95]. Despite substantial community-led programs, large gaps remain in resources, funding, and coverage to scale-up services.

### Strengths and limitations

This review uniquely builds upon the literature and contributes to our understanding of multilevel factors that potentiate and mitigate STBBI risk among cis women, cis men, and trans sex worker populations. To the best of our knowledge, this is the first comprehensive synthesis of determinants and intervention and prevention strategies to reduce STBBI burden among sex workers of all genders, specific to high-income countries. The majority of research and data on STBBIs among sex workers are largely confined to LMICs, with a dearth of research focusing on the experiences of cis men and trans sex workers. Studies seldom disaggregate data by sex work involvement [115], which limited our ability to understand the determinants of, and interventions for, STBBIs in these populations. Given the heterogeneity of sex worker populations, and distinct vulnerabilities faced by cis men and trans sex workers, further research utilizing mixed-methods should be implemented to delineate the intersections of risk and ameliorate critical health inequalities for all sex workers.

## Conclusions

The high burden of STBBIs among sex workers across high-income settings is of major concern. This review highlights promising strategies that need to be scaled up and evaluated to prevent STBBIs among sex workers. Research and evidence suggest that structural and community/sex worker-led approaches are paramount to addressing the high STBBI burden and gaps in access to care and are necessary to realizing health and human rights for sex workers.

## Abbreviations

ART: Antiretroviral therapy
HCV: Hepatitis C virus
HIV: Human immunodeficiency virus
LMICs: Lower and middle-income countries
MSM: Men who have sex with men
MSW: Male sex worker
NSWP: Global Network of Sex Work Projects
PCEPA: Protection of Communities and Exploited Persons Act
PrEP: Pre-exposure prophylaxis
STBBI: Sexually transmitted and blood borne infection
STI: Sexually transmitted infection
UNAIDS: United Nations Programme on HIV/AIDS
UNDP: United Nations Development Program
UNFPA: United Nations Population Fund
WHO: World Health Organization

## References

[1] Beyrer C, Crago AL, Bekker LG, Butler J, Shannon K, Kerrigan D (2015). An action agenda for HIV and sex workers. Lancet.

[2] Shannon K, Strathdee SA, Goldenberg SM, Duff P, Mwangi P, Rusakova M (2015). Global epidemiology of HIV among female sex workers: influence of structural determinants. Lancet.

[3] Baral S, Beyrer C, Muessig K, Poteat T, Wirtz AL, Decker MR (2012). Burden of HIV among female sex workers in low-income and middle-income countries: a systematic review and meta-analysis. Lancet Infect Dis.

[4] Beyrer C, Crago AL, Bekker LG, Butler J, Shannon K, Kerrigan D (2015). The lancet supplementary appendix. Lancet.

[5] Sullivan PS, Jones JS, Baral SD (2014). The global north: HIV epidemiology in high-income countries. Curr Opin HIV AIDS.

[6] Goldenberg SM, Duff P, Krusi A (2015). Work environments and HIV prevention: a qualitative review and meta-synthesis of sex worker narratives. BMC Public Health.

[7] Baral SD, Friedman MR, Geibel S, Rebe K, Bozhinov B, Diouf D (2015). Male sex workers: practices, contexts, and vulnerabilities for HIV acquisition and transmission. Lancet.

[8] Poteat T, Wirtz AL, Radix A, Borquez A, Silva-Santisteban A, Deutsch MB (2015). HIV risk and preventive interventions in transgender women sex workers. Lancet.

[9] Fieland K, Walters K, Simoni J, Meyer I, Northridge M (2007). Determinants of health among two-spirit American Indians and Alaska natives. The health of sexual minorities.

[10] Ristock J, Zoccole A, Passante L. Aboriginal two-spirit and LGBTQ migration, mobility and health research project: final report. Winnipeg, MB; 2010. http://www.2spirits.com/PDFolder/MMHReport.pdf.

[11] World Health Organization. Prevention and Treatment of HIV and Other Sexually Transmitted Infections for Sex Workers in Low- and Middle-Income Countries. Geneva; 2012. http://www.who.int/hiv/pub/guidelines/sex_worker/en/index.html. Accssed 24 July 2017. 26131541

[12] Argento E, Reza-Paul S, Lorway R, Jain J, Bhagya M, Fathima M (2011). Confronting structural violence in sex work: lessons from a community-led HIV prevention project in Mysore, India. AIDS Care.

[13] Kerrigan D, Kennedy CE, Morgan-Thomas R, Reza-Paul S, Mwangi P, Win KT (2015). A community empowerment approach to the HIV response among sex workers: effectiveness, challenges, and considerations for implementation and scale-up. Lancet.

[14] Aggleton P, Wood K, Malcolm A, Parker R. HIV-related stigma, Discrimination and Human Rights Violations: Case Studies of Successful Programmes. Geneva; 2005. http://data.unaids.org/UNA-docs/JC999-HRViolations_en.pdf. Accessed 24 July 2017.

[15] Global Commission on HIV and the Law. Risks, Rights & Health. New York; 2012. http://www.hivlawcommission.org/. Accessed 24 July 2017.

[16] Amnesty International. Sex Worker’s rights are human rights. 2015. https://www.amnesty.org/en/latest/news/2015/08/sex-workers-rights-are-human-rights/. Accessed 24 July 2017.

[17] Ferris S (2015). Street sex work and Canadian cities: resisting a dangerous order.

[18] Shannon K, Goldenberg SM, Deering KN, Strathdee S (2014). HIV infection among female sex workers in concentrated and high prevalence epidemics: why a structural determinants framework is needed. Curr Opin HIV AIDS.

[19] Rhodes T (2009). Risk environments and drug harms: a social science for harm reduction approach. Int J Drug Policy..

[20] World Bank. Data & Statistics. Washington, DC; 2016. https://data.worldbank.org/. Accessed 03 Aug 2017.

[21] Sethi G, Holden BM, Gaffney J, Greene L, Ghani AC, Ward H (2006). HIV, sexually transmitted infections, and risk behaviours in male sex workers in London over a 10 year period. Sex Transm Infect.

[22] Fournet N, Koedijk FDH, van Leeuwen AP, van Rooijen MS, van der Sande MAB, van Veen MG (2016). Young male sex workers are at high risk for sexually transmitted infections, a cross-sectional study from Dutch STI clinics, the Netherlands, 2006–2012. BMC Infect Dis.

[23] Clements-Nolle K, Guzman R, Harris SG (2008). Sex trade in a male-to-female transgender population: psychosocial correlates of inconsistent condom use. Sex Health.

[24] Nemoto T, Bödeker B, Iwamoto M, Sakata M (2014). Practices of receptive and insertive anal sex among transgender women in relation to partner types, sociocultural factors, and background variables. AIDS Care.

[25] Shannon K, Bright V, Gibson K, Tyndall MW, Maka Project Partnership (2007). Sexual and drug-related vulnerabilities for HIV infection among women engaged in sex work in Vancouver, Canada. Can J Public Heal.

[26] Platt L, Jolley E, Rhodes T, Hope V, Latypov A, Reynolds L, et al. Factors mediating HIV risk among female sex workers in Europe: a systematic review and ecological analysis. BMJ Open. 2013;3:e002836. 10.1136/bmjopen-2013-002836 PMC3731729 23883879

[27] Abad N, Baack BN, O’Leary A, Mizuno Y, Herbst JH, Lyles CM (2015). A systematic review of HIV and STI behavior change interventions for female sex workers in the United States. AIDS Behav.

[28] Kerr T, Shannon K, Ti L, Strathdee S, Hayashi K, Nguyen P (2016). Sex work and HIV incidence among people who inject drugs. AIDS.

[29] Argento E, Muldoon K, Duff P, Simo A, Deering K, Shannon K (2014). High prevalence and partner correlates of physical and sexual violence by intimate partners among street and off-street sex workers. PLoS One.

[30] Platt L, Grenfell P, Bonell C, Creighton S, Wellings K, Parry J (2011). Risk of sexually transmitted infections and violence among indoor-working female sex workers in London: the effect of migration from Eastern Europe. Sex Transm Infect.

[31] Goldenberg SM, Chettiar J, Simo A, Silverman JG, Strathdee SA, Montaner JSG (2014). Early sex work initiation independently elevates odds of HIV infection and police arrest among adult sex workers in a Canadian setting. J Acquir Immune Defic Syndr.

[32] Duff P, Tyndall M, Buxton J, Zhang R, Kerr T, Shannon K. Sex-for-crack exchanges: associations with risky sexual and drug use niches in an urban Canadian city. Harm Reduct J. 2013;10:e2010008. 10.1186/1477-7517-10-29. 10.1186/1477-7517-10-29 PMC3833173 24238367

[33] Wood E, Schachar J, Li K, Stoltz JA, Shannon K, Miller C (2007). Sex trade involvement is associated with elevated HIV incidence among injection drug users in Vancouver. Addict Res Theory.

[34] Harawa NT, Bingham TA (2009). Exploring HIV prevention utilization among female sex workers and male-to-female transgenders. AIDS Educ Prev.

[35] Surratt HL, Kurtz SP (2012). Foster care history and HIV infection among drug-using African American female sex workers. AIDS Behav.

[36] Lee J, Jung S-Y, Kwon DS, Jung M, Park B-J. Condom use and prevalence of genital chlamydia trachomatis among the Korean female sex workers. Epidemiol Health. 2010;32. 10.4178/epih/e2010008 PMC2984866 21191461

[37] Croxford S, Platt L, Hope VD, Cullen KJ, Parry JV, Ncube F (2015). Sex work amongst people who inject drugs in England, Wales and Northern Ireland: findings from a national survey of health harms and behaviours. Int J Drug Policy.

[38] Goldenberg S, Deering K, Nguyen P, Dobrer S, Socias M, Guillemi S, et al. Elevated hepatitis C incidence among youth, women co-infected with HIV/STIs and sex workers who use crack cocaine in Vancouver, Canada: gaps and opportunities for HCV prevention and treatment. In: International AIDS Society Conference. Vancouver; 2015.

[39] Kweon S-S, Shin M-H, Song H-J, Jeon D-Y, Choi J-S (2006). Seroprevalence and risk factors for hepatitis C virus infection among female commercial sex workers in South Korea who are not intravenous drug users. Am J Trop Med Hyg.

[40] Dias S, Gama A, Fuertes R, Mendão L, Barros H (2015). Risk-taking behaviours and HIV infection among sex workers in Portugal: results from a cross-sectional survey. Sex Transm Infect.

[41] Operario D, Soma T, Underhill K (2008). Sex work and HIV status among transgender women - systematic review and meta-analysis. J Acquir Immune Defic Syndr.

[42] Bauer G, Travers R, Scanlon K, Coleman T (2012). High heterogeneity of HIV-related sexual risk among transgender people in Ontario, Canada: a province-wide respondent-driven sampling survey. BMC Public Health.

[43] Verhaegh-Haasnoot A, Dukers-Muijrers NHTM, Hoebe CJPA (2015). High burden of STI and HIV in male sex workers working as internet escorts for men in an observational study: a hidden key population compared with female sex workers and other men who have sex with men. BMC Infect Dis.

[44] Bacon O, Lum P, Hahn J, Evans J, Davidson P, Moss A (2006). Commercial sex work and risk of HIV infection among young drug-injecting men who have sex with men in San Francisco. Sex Transm Dis.

[45] Mimiaga MJ, Reisner SL, Tinsley JP, Mayer KH, Safren SA (2008). Street workers and internet escorts: contextual and psychosocial factors surrounding HIV risk behavior among men who engage in sex work with other men. J Urban Heal..

[46] Timpson SC, Ross MW, Williams ML, Atkinson J (2007). Characteristics, drug use, and sex partners of a sample of male sex workers. Am J Drug Alcohol Abuse.

[47] Prestage G, Jin F, Bavinton B, Hurley M (2014). Sex workers and their clients among australian gay and bisexual men. AIDS Behav.

[48] Myers T, Allman D, Xu K, Remis RS, Aguinaldo J, Burchell A (2009). The prevalence and correlates of hepatitis C virus (HCV) infection and HCV-HIV co-infection in a community sample of gay and bisexual men. Int J Infect Dis.

[49] Surratt HL, Kurtz SP, Chen M, Mooss A (2012). HIV risk among female sex workers in Miami: the impact of violent victimization and untreated mental illness. AIDS Care.

[50] Romero-Daza N, Weeks M, Singer M (2005). Conceptualizing the impact of indirect violence on HIV risk among women involved in street-level prostitution. Aggress Violent Behav.

[51] Reisner SL, Mimiaga MJ, Mayer KH, Tinsley JP, Safren SA (2008). Tricks of the trade: sexual health behaviors, the context of HIV risk, and potential prevention intervention strategies for male sex workers. J LGBT Health Res.

[52] Grov C, Rodríguez-Díaz CE, Jovet-Toledo GG, Parsons JT (2015). Comparing male escorts’ sexual behaviour with their last male client versus non-commercial male partner. Cult Health Sex..

[53] Herbst JH, Jacobs ED, Finlayson TJ, McKleroy VS, Neumann MS, Crepaz N (2008). Estimating HIV prevalence and risk behaviors of transgender persons in the United States: a systematic review. AIDS Behav.

[54] Clements-Nolle K, Guzman R, Harris S (2008). Sex trade in a male-to-female transgender population: psychosocial correlates of inconsistent condom use. Sex Health.

[55] Nemoto T, Boedeker B, Iwamoto M (2011). Social support, exposure to violence and transphobia, and correlates of depression among male-to-female transgender women with a history of sex work. Am J Public Health.

[56] Deering K, Amin A, Shoveller J, Nesbitt A, Garcia-Moreno C, Duff P (2014). A systematic review of the global magnitude and drivers of violence against sex workers. Am J Public Health.

[57] Shannon K, Csete J (2010). Violence, condom negotiation, and HIV/STI risk among sex workers. JAMA.

[58] Lazarus L, Chettiar J, Deering K, Nabess R, Shannon K (2011). Risky health environments: women sex workers’ struggles to find safe, secure and non-exploitative housing in Canada’s poorest postal code. Soc Sci Med.

[59] Platt L, Grenfell P, Fletcher A, Sorhaindo A, Jolley E, Rhodes T (2013). Systematic review examining differences in HIV, sexually transmitted infections and health-related harms between migrant and non-migrant female sex workers. Sex Transm Infect.

[60] Shannon K, Strathdee SA, Shoveller J, Rusch M, Kerr T, Tyndall MW (2009). Structural and environmental barriers to condom use negotiation with clients among female sex workers: implications for HIV-prevention strategies and policy. Am J Public Health.

[61] Argento E, Duff P, Bingham B, Chapman J, Nguyen P, Strathdee S, et al. Social cohesion among sex workers and client condom refusal in a Canadian setting: implications for structural and community-led interventions. AIDS Behav. 2016;20:1275-83. 10.1007/s10461-015-1230-8 PMC4846583 26499335

[62] Oppal WT. Forsaken: the report of the missing women Commission of Inquiry. 2012. http://www.missingwomeninquiry.ca/wp-content/uploads/2010/10/Forsaken-ES-web-RGB.pdf. Accessed 24 July 2017.

[63] Shannon K, Kerr T, Strathdee S, Shoveller J, Montaner J, Tyndall M. Prevalence and structural correlates of gender based violence among a prospective cohort of female sex workers. BMJ. 2009;339:b2939. 10.1136/bmj.b2939 PMC2725271 19671935

[64] Shannon K, Rusch M, Shoveller J, Alexson D, Gibson K, Tyndall M (2008). Mapping violence and policing as an environmental-structural barrier to health service and syringe availability among substance-using women in street-level sex work. Int J Drug Policy..

[65] Benoit C, Carroll D, Chaudhry M (2003). In search of a healing place: aboriginal women in Vancouver’s downtown eastside. Soc Sci Med.

[66] Open Society Foundations. Criminalizing condoms: How policing practices put sex workers and HIV services at risk in Kenya, Namibia, Russia, South Africa, USA, and Zimbabwe. New York; 2012.

[67] Decker MR, Crago AL, Chu SKH, Sherman SG, Seshu MS, Buthelezi K (2015). Human rights violations against sex workers: burden and effect on HIV. Lancet.

[68] Csete J, Cohen J (2010). Health benefits of legal services for criminalized populations: the case of people who use drugs, sex workers and sexual and gender minorities. J Law, Med Ethics.

[69] Bruckert C, Hannem S (2013). Rethinking the prostitution debates: transcending structural stigma in systemic responses to sex work. Can J Law Soc.

[70] Shannon K, Kerr T, Allinott S, Chettiar J, Shoveller J, Tyndall MW (2008). Social and structural violence and power relations in mitigating HIV risk of drug-using women in survival sex work. Soc Sci Med.

[71] Krüsi A, Pacey K, Bird L, Taylor C, Chettiar J, Allan S, et al. Criminalisation of clients: reproducing vulnerabilities for violence and poor health among street-based sex workers in Canada-a qualitative study. BMJ Open. 2014;4. 10.1136/bmjopen-2014-005191 PMC4054637 24889853

[72] Supreme Court of Canada (2013). Canada (Attorney General) vs. Bedford.

[73] Lazarus L, Deering KN, Nabess R, Gibson K, Tyndall MW, Shannon K (2012). Occupational stigma as a primary barrier to health care for street-based sex workers in Canada. Cult Health Sex..

[74] Underhill K, Morrow KM, Colleran C, Holcomb R, Calabrese SK, Operario D (2015). A qualitative study of medical mistrust, perceived discrimination, and risk behavior disclosure to clinicians by U.S. male sex workers and other men who have sex with men: implications for biomedical HIV prevention. J Urban Heal..

[75] Whitaker T, Ryan P, Cox G (2011). Stigmatization among drug-using sex workers accessing support services in Dublin. Qual Health Res.

[76] Krüsi A, Chettiar J, Ridgway A, Abbott J, Strathdee SA, Shannon K (2012). Negotiating safety and sexual risk reduction with clients in unsanctioned safer indoor sex work environments: a qualitative study. Am J Public Health.

[77] Deering K, Lyons T, Feng C, Nosyk B, Strathdee S, Montaner J (2013). Client demands for unsafe sex: the socioeconomic risk environment for HIV among street and off-street sex workers. J Acquir Immune Defic Syndr.

[78] Lyons T, Krusi A, Pierre L, Kerr T, Small W, Shannon K. Negotiating violence in the context of transphobia and criminalization: The experiences of trans sex workers in Vancouver, Canada. Qual Health Res. 2015;27:182–90. 10.1177/1049732315613311 PMC4848175 26515922

[79] Socias ME, Marshall BDL, Aristegui I, Romero M, Cahn P, Kerr T (2014). Factors associated with healthcare avoidance among transgender women in Argentina. Int J Equity Health.

[80] Sausa LA, Keatley J, Operario D (2007). Perceived risks and benefits of sex work among transgender women of color in San Francisco. Arch Sex Behav.

[81] Matthen P, Lyons T, Taylor M, Jollimore J, Jennex J, Shannon K. “I walked into the industry for survival and came out of a closet”: how gender and sexual identities shape sex work experiences among men, two spirit, and trans individuals in Vancouver. Men Masc. 2018;21:479–500. PMC6326376 30636865

[82] Goldenberg SM, Liu V, Nguyen P, Chettiar J, Shannon K. International Migration from Non-endemic Settings as a Protective Factor for HIV/STI Risk Among Female Sex Workers in Vancouver, Canada. J Immigr Minor Heal. 2015;27:182–90. 10.1007/s10903-014-0011-1 PMC4185267 24700025

[83] Mc Grath-Lone L, Marsh K, Hughes G, Ward H (2014). The sexual health of female sex workers compared with other women in England: analysis of cross-sectional data from genitourinary medicine clinics. Sex Transm Infect.

[84] Deering KN, Montaner JS, Chettiar J, Jia J, Ogilvie G, Buchner C (2015). Successes and gaps in uptake of regular, voluntary HIV testing for hidden street- and off-street sex workers in Vancouver. Canada AIDS Care.

[85] Sou J, Shannon K, Li J, Nguyen P, Strathdee SA, Shoveller J (2015). Structural determinants of inconsistent condom use with clients among migrant sex workers. Sex Transm Dis.

[86] Anderson S, Jia JX, Liu V, Chattier J, Krüsi A, Allan S (2015). Violence prevention and municipal licensing of indoor sex work venues in the greater Vancouver area: narratives of migrant sex workers, managers and business owners. Cult Health Sex.

[87] Goldenberg SM, Montaner J, Duff P, Nguyen P, Dobrer S, Guillemi S, et al. Structural barriers to antiretroviral therapy among sex workers living with HIV: findings of a longitudinal study in Vancouver, Canada AIDS Behav 2015. doi:10.1007/s10461-015-1102-2. 10.1007/s10461-015-1102-2 PMC4704989 26148850

[88] Mc Grath-Lone L, Marsh K, Hughes G, Ward H (2014). The sexual health of male sex workers in England: analysis of cross-sectional data from genitourinary medicine clinics. Sex Transm Infect.

[89] Goldenberg SM, Chettiar J, Nguyen P, Dobrer S, Montaner J, Shannon K (2014). Complexities of short-term mobility for sex work and migration among sex workers: violence and sexual risks, barriers to care, and enhanced social and economic opportunities. J Urban Heal.

[90] Mountain E, Mishra S, Vickerman P, Pickles M, Gilks C, Boily MC. Antiretroviral therapy uptake, attrition, adherence and outcomes among hiv-infected female sex workers: a systematic review and meta-analysis. PLoS One. 2014;9:e105645. 10.1371/journal.pone.0105645 PMC4179256 25265158

[91] Socías ME, Mph KS, Montaner JS, Guillemi S, Ma SD, Nguyen P (2015). Gaps in the hepatitis C continuum of care among sex workers in Vancouver, British Columbia: implications for voluntary hepatitis C virus testing, treatment and care. Can J Gastroenterol Hepatol.

[92] Kerrigan D, Wirtz A, Baral S, Decker M, Murray L, Poteat T (2013). The global HIV epidemics among sex workers.

[93] Jana S, Basu I, Rotheram-Borus MJ, Newman PA (2004). The Sonagachi Project: a sustainable community intervention program. AIDS Educ Prev.

[94] Reza-Paul S, Lorway R, O’Brien N, Lazarus L, Jain J, Bhagya M (2012). Sex worker-led structural interventions in India: a case study on addressing violence in HIV prevention through the Ashodaya Samithi collective in Mysore. Indian J Med Res.

[95] NSWP (Network of Sex Work Projects). St James Infirmary posters presented at international conference. Global Network of Sex Work Projects. 2012. https://www.nswp.org/. Accessed 03 Aug 2017.

[96] Cohan D, Lutnick A, Davidson P, Cloniger C, Herlyn A, Breyer J (2006). Sex worker health: San Francisco style. Sex Transm Infect.

[97] Deering KN, Kerr T, Tyndall MW, Montaner JSG, Gibson K, Irons L (2011). A peer-led mobile outreach program and increased utilization of detoxification and residential drug treatment among female sex workers who use drugs in a Canadian setting. Drug Alcohol Depend.

[98] Kim SR, Goldenberg SM, Duff P, Nguyen P, Gibson K, Shannon K (2015). Uptake of a women-only, sex-work-specific drop-in center and links with sexual and reproductive health care for sex workers. Int J Gynecol Obstet.

[99] Deering K, Shannon K, Sinclair H, Parsad D, Gilbert E, Tyndall M (2009). Piloting a peer-driven intervention model to increase access and adherence to antiretroviral therapy and HIV care among street-entrenched HIV-positive women in Vancouver. AIDS Patient Care STDs.

[100] Abel GM, Fitzgerald LJ, Brunton C (2009). The impact of decriminalisation on the number of sex Workers in new Zealand. J Soc Policy.

[101] Abel GM, Fitzgerald LJ (2012). “The street”s got its advantages’: movement between sectors of the sex industry in a decriminalised environment. Health Risk Soc.

[102] Government of Canada Department of Justice. Technical Paper: Bill C-36, Protection of Communities and Exploited Persons Act. Ottawa, ON; 2015. http://www.justice.gc.ca/eng/rp-pr/other-autre/protect/p1.html. Accessed 24 July 2017.

[103] Duff P, Ogilvie G, Shoveller J, Amram O, Chettiar J, Nguyen P (2016). Barriers to cervical screening among sex Workers in Vancouver. Am J Public Health.

[104] Duff P, Shoveller J, Dobrer S, Ogilvie G, Montaner J, Chettiar J, et al. The relationship between social, policy and physical venue features and social cohesion on condom use for pregnancy prevention among sex workers: A safer indoor work environment scale. J Epidemiol Community Heal. 2015;:1–7. doi:10.1136/jech-2014-204427. 10.1136/jech-2014-204427 PMC4675653 25678713

[105] Minichiello V, Scott J, Callander D. A new public health context to understand male sex work. BMC Public Health. 2015;15:1–11. 10.1186/s12889-015-1498-7 PMC4419468 25879716

[106] Argento E, Taylor M, Jollimore J, Taylor C, Jennex J, Krusi A, et al. The loss of Boystown and transition to online sex work: strategies and barriers to increase safety among men sex workers and clients of men. Amercian J Men’s Heal. 2016. 10.1177/1557988316655785. 10.1177/1557988316655785 PMC5484742 27352925

[107] Ballester-Arnal R, Gil-Llario MD, Salmeron-Sanchez P, Gimenez-Garcia C (2014). HIV prevention interventions for young male commercial sex workers. Curr HIV/AIDS Rep.

[108] Williams M, Bowen A, Timpson S, Ross M, Atkinson J (2006). HIV prevention and street-based male sex workers: an evaluation of brief interventions. AIDS Educ Prev.

[109] Parsons J, Koken J, Bimbi D (2007). Looking beyond HIV: eliciting individual and community needs of male internet escorts. J Homosex.

[110] Rhodes T, Wagner K, Strathdee S, Shannon K, Davidson P, Bourgois P, O’Campo P, Dunn J (2012). Structural violence and structural vulnerability within the risk environment: theoretical and methodological perspectives for a social epidemiology of HIV risk among IDU and SW. Rethinking social epidemiology: towards a science of change.

[111] Shannon K, Montaner JSG (2012). The politics and policies of HIV prevention in sex work. Lancet Infect Dis.

[112] Jeal N, Macleod J, Turner K, Salisbury C (2015). Systematic review of interventions to reduce illicit drug use in female drug-dependent street sex workers. BMJ Open.

[113] Surratt HL, Inciardi JA (2010). An effective HIV risk-reduction protocol for drug-using female sex workers. J Prev Interv Community.

[114] NSWP (Network of Sex Work Projects). HIV and STI Testing and Treatment Policies. 2016. http://www.nswp.org/resource/community-guide-hiv-and-sti-testing-and-treatment-policies. Accessed 24 July 2017.

[115] Baral SD, Poteat T, Stromdahl S, Wirtz AL, Guadamuz TE, Beyrer C (2013). Worldwide burden of HIV in transgender women: a systematic review and meta-analysis. Lancet Infect Dis.

